# Jagged-1 Signaling in the Bone Marrow Microenvironment Promotes Endothelial Progenitor Cell Expansion and Commitment of CD133^+^ Human Cord Blood Cells for Postnatal Vasculogenesis

**DOI:** 10.1371/journal.pone.0166660

**Published:** 2016-11-15

**Authors:** Mika Ishige-Wada, Sang-Mo Kwon, Masamichi Eguchi, Katsuto Hozumi, Hideki Iwaguro, Taro Matsumoto, Noboru Fukuda, Hideo Mugishima, Haruchika Masuda, Takayuki Asahara

**Affiliations:** 1 Department of Regenerative Medicine Science, Tokai University School of Medicine, Isehara, Japan; 2 Department of Pediatrics and Child Health, Nihon University school of Medicine, Tokyo, Japan; 3 Department of Immunology, Tokai University School of Medicine, Isehara, Japan; 4 Division of Cell Regeneration and Transplantation, Nihon University School of Medicine, Tokyo, Japan; 5 Division of Nephrology Hypertension and Endocrinology, Department of Medicine, Nihon University School of Medicine, Tokyo, Japan; University of Alabama at Birmingham, UNITED STATES

## Abstract

Notch signaling is involved in cell fate decisions during murine vascular development and hematopoiesis in the microenvironment of bone marrow. To investigate the close relationship between hematopoietic stem cells and human endothelial progenitor cells (EPCs) in the bone marrow niche, we examined the effects of Notch signals [Jagged-1 and Delta-like ligand (Dll)-1] on the proliferation and differentiation of human CD133^+^ cell-derived EPCs. We established stromal systems using HESS-5 murine bone marrow cells transfected with human *Jagged-1* (hJagged-1) or human *Dll-1* (hDll-1). CD133^+^ cord blood cells were co-cultured with the stromal cells for 7 days, and then their proliferation, differentiation, and EPC colony formation was evaluated. We found that hJagged-1 induced the proliferation and differentiation of CD133^+^ cord blood EPCs. In contrast, hDll-1 had little effect. CD133^+^ cells stimulated by hJagged-1 differentiated into CD31^+^/KDR^+^ cells, expressed vascular endothelial growth factor-A, and showed enhanced EPC colony formation compared with CD133^+^ cells stimulated by hDll-1. To evaluate the angiogenic properties of hJagged-1- and hDll-1-stimulated EPCs *in vivo*, we transplanted these cells into the ischemic hindlimbs of nude mice. Transplantation of EPCs stimulated by hJagged-1, but not hDll-1, increased regional blood flow and capillary density in ischemic hindlimb muscles. This is the first study to show that human Notch signaling influences EPC proliferation and differentiation in the bone marrow microenvironment. Human Jagged-1 induced the proliferation and differentiation of CD133^+^ cord blood progenitors compared with hDll-1. Thus, hJagged-1 signaling in the bone marrow niche may be used to expand EPCs for therapeutic angiogenesis.

## Introduction

Notch signaling plays a crucial role in cell fate determination of a variety of cell types during development and postnatal tissue organization, including murine vascular development and angiogenesis [1, 2]. Mutations of Notch receptors and ligands in mice cause abnormal organization of vascular and hematopoietic systems with severe hemorrhaging, which is embryonic lethal in the Notch signaling null mouse [3]. Two human diseases, cerebral autosomal dominant arteriopathy with subcortical infarcts and leukoencephalopathy (CADSIL) and Alagille Syndrome exhibit vascular system abnormalities caused by Notch pathway mutations [4, 5]. Notch signaling is initiated by interactions between Notch receptors and their ligands expressed on cells. Mammals express four Notch receptors (Notch 1–4) and five Notch ligands [Jagged-1, -2, and Delta-like ligand (Dll)-1, -2, and -4]. Interactions of Notch receptors with the membrane-bound ligands of Delta and Jagged gene families are critical for Notch activation. Ligand binding induces γ-secretase-mediated cleavage and translocation of the Notch intracellular domain into the nucleus where it interacts with DNA-binding protein RBP-Jk to induce downstream target genes [1]. Primitive human CD34^+^ bone marrow cells express all Notch receptors [6]. Furthermore, primary cells and cultured stromal cells derived from the aorta-gonad-mesonephros, fetal liver, bone marrow, and osteoblasts express Jagged-1, Dll-1, and Dll-4 [7–9]. Therefore, these ligands expressed on stromal cells might interact with their respective Notch receptors on primitive hematopoietic cells in hematopoietic stem cell niches.

Endothelial progenitor cells (EPCs) identified as CD34^+^ mononuclear cells isolated from adult peripheral blood are a functional angiogenetic modulator of postnatal neovascularization [10]. Peripheral blood EPCs were originally derived from bone marrow, but are also present in cord blood (CB) as CD34^+^ and CD133^+^ cells [11]. Cytokines induce EPCs to proliferate and differentiate in the bone marrow, mobilize to systemic circulation, migrate to ischemic sites, differentiate into mature endothelial cells, and secrete angiogenic factors [12–14]. In the bone marrow microenvironment, the quantitative and qualitative features of EPCs might be regulated by several molecular mechanisms, which is similar to hematopoietic stem cells. We have demonstrated that Jagged-1 in the bone marrow niche is required for EPC development of neovascularization in mice [15]. Furthermore, Jagged-1 and Notch1 are critical for the role of EPCs in a mouse injury model [16, 17]. However, little is known about human EPCs and their development in the bone marrow niche.

In this study, we established an *in vitro* co-culture system similar to the bone marrow niche using HESS-5 bone marrow stromal cells to investigate the functional importance of Notch signals for human EPC-mediated neovascularization and the proliferation and differentiation of human CB-derived EPCs *in vitro* and *in vivo*.

## Materials and Methods

### CD133^+^ cell preparation

Human umbilical CB samples (50–100 mL) were collected in sterile blood packs (SC-200; Terumo Corp., Tokyo, Japan) containing a citrate-dextrose solution as the anticoagulant. Written informed consent was obtained from all mothers before labor and delivery. Protocols for sampling human umbilical CB were approved by our Institutional Review Board, the Clinical Investigation Committee at Tokai University School of Medicine. Mononuclear cells were separated by Ficoll-Hypaque density gradient centrifugation. The mononuclear cell layer was collected, washed twice with 2 mM ethylenediaminetetraacetic acid (EDTA) in PBS, and resuspended in degassed PBS with 0.5% bovine serum albumin (BSA) and 2 mM EDTA. CD133^+^ CB cells were separated from 1 × 10^8^ mononuclear cells by a magnetic bead separation method (Miltenyi Biotec, Gladbach, Germany). In brief, CD133^+^ cells were labeled with a hapten-conjugated monoclonal antibody (mAb) against human CD133 (clone AC133: Miltenyi Biotec), followed by microbeads coupled with an anti-hapten mAb. The bead-positive cells were enriched on positive selection columns twice in a magnetic field. Flow cytometric analysis of purified cells using a phycoerythrin (PE)-conjugated anti-CD133 mAb of a different clone (clone 293C3; Miltenyi Biotec) showed that 95% of the selected cells were positive for CD133.

### Flow cytometry analysis

Flow cytometric profiles were obtained with a FACSCalibur flow cytometer and CellQuest software (Becton Dickinson Immunocytometry Systems, Mountain View, CA). Briefly, 1 × 10^4^ magnetically separated CD133^+^ CB cells or 1 × 10^5^ cultured CB cells collected from each transfected HESS-5 stroma were washed twice with PBS containing 1% fraction V BSA (Sigma, St Louis, MO), preincubated with 10 μL FcR blocking regent (Miltenyi Biotec) to block nonspecific binding for 30 minutes at 4°C, and then incubated with mAbs for 30 minutes at 4°C. For kinase-insert domain containing receptor (KDR) staining, biotinated anti-mouse IgG_1_ (BD Biosciences, San Jose, CA) and allophycocyanin (APC)-conjugated streptavidin (BD Biosciences) were used as secondary and tertiary antibodies. The stained cells were washed three times with PBS containing 1% BSA, resuspended in 0.5 mL PBS/1% BSA/propidium iodide (PI; Sigma), and analyzed by the FACSCalibur flow cytometer. Dead cells were excluded from the plots based on positive PI staining. The following mAbs were used to characterize the cultured CD133^+^ CB cells: CD34-fluorescein-5-isothiocyanate (FITC) (clone 581), CD34-PE (clone 581), CD45-FITC (clone HI30), CD31-FITC (clone WM59) (BD Biosciences), AC133-APC (clone AC133) (Miltenyi Biotec), and KDR (Sigma). For negative isotype controls, mouse IgG_1_-FITC (clone MOPC-21), mouse IgG_1_-PE (clone MOPC-21), mouse IgG_1_-APC (clone MOPC-21), and unlabeled mouse IgG_1_ (clone MOPC-21) (BD Biosciences) were used.

### HESS-5 cell line

We used the murine bone marrow-derived stromal cell line HESS-5 (provided by Dr. K. Ando, Tokai University, Kanagawa, Japan), which maintains the reconstitution ability of *ex vivo*-generated human hematopoietic stem cells and does not express *Jagged-1* or *Dll-1* [18]. Originally, HESS-5 cells were grown in minimal essential medium (MEM; Gibco, Grand Island, NY) supplemented with 10% horse serum (Gibco) and penicillin/streptomycin (Gibco).

### Retroviruses and producer cell lines

**Fig 1:**
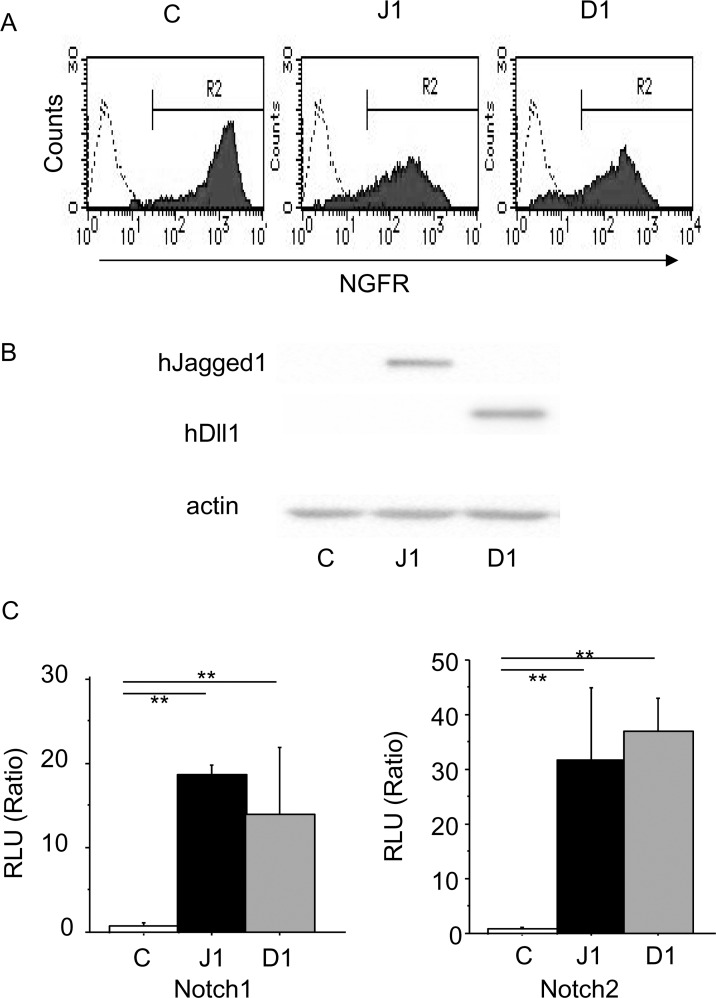
Expression and promoter activity of Notch signals in HESS-5 cells transfected with Notch ligand genes. (A) Flow cytometric analysis of NGFR expression in control vector- (C), human *Jagged-1* (J1)- and human *Dll-1* (D1)-transfected HESS-5 cells, and the sorting gate (R2) for NGFR^+^ transfected cells. (B) Western blot analysis of hJagged-1 and hDll-1 proteins in HESS-5 cells. Actin was used as an internal control. (C) Notch1 and Notch2 reporter cell lines transfected with RBP-Jk-luc were co-cultured with HESS-5 stromal cells. Luciferase activity (relative light units) was normalized to the activity of Renilla luciferase. Data are expressed as means ± standard deviation (SD) (n = 3). *P < 0.01 between the indicated values.

We established three types of feeder cells: control (HESS-5 cells transfected with an empty vector), hJagged-1 (HESS-5 cells transfected with human *Jagged-1*), and hDll-1(HESS-5 cells transfected with human *Dll-1*). Full-length cDNAs encoding human *Jagged-1* and *Dll-1* (provided by Dr. K. Hozumi and Dr. G. Ando, Tokai University, Kanagawa, Japan) were cloned into the *Not*I and *Xho*I restriction sites of GCDNsamI/N retrovirus vectors. These vectors were transfected transiently into PLAT-E packaging cells (provided by Dr. T. Kitamura, The University of Tokyo, Tokyo, Japan) using Lipofectamine transfection reagent (Invitrogen, Carlsbad, CA). The culture supernatants were harvested as the source of the retrovirus as described previously [19]. HESS-5 cells were grown in MEM supplemented with 10% horse serum and penicillin/streptomycin. The day before transfections, 5 × 10^3^ HESS-5 cells were seeded in a 24-well plate. The cells were then incubated in 0.1 mL of retroviral supernatant containing either human *Jagged-1*, human *Dll-1*, or empty viruses in the presence of 8 μg/mL polybrene (Sigma) for 16 hours at 37°C with 5% CO_2_. The transfection mixture was removed, α-MEM with 10% horse serum was added, and the cells were cultured for 24 hours. Then, the cells were transferred to a 6-well plate for further culture in α-MEM with 10% horse serum and penicillin/streptomycin. At 48 hours after culture, the transfected HESS-5 cells were stained with human nerve growth factor receptor (NGFR)-PE (clone CD40-1457) (BD Biosciences) and analyzed by flow cytometry (Fig 1A). The transduced cells were subsequently sorted using a FACS Vantage cell sorter (Becton Dickinson Immunocytometry Systems). More than 99% of the cells expressed NGFR, which were used for co-culture experiments with CB CD133^+^ cells.

### Western blotting

For western blot analysis, cell membrane proteins prepared from cultured retroviral transduced HESS-5 cells were separated on a 7.5% polyacrylamide gel (10 μg protein/lane). The proteins were transferred to nitrocellulose membranes (Hybond-ECL; Amersham Pharmacia Biotech, Piscataway, NJ), and incubated overnight at 4°C with primary antibodies: goat polyclonal IgG against human Jagged-1 (C-20) (Santa Cruz Biotechnology, Santa Cruz, CA), rabbit polyclonal IgG against human Dll-1 (H-265) (Santa Cruz Biotechnology), or rabbit polyclonal IgG against actin (Sigma). The membranes were washed three times and incubated with horseradish peroxidase-conjugated donkey anti-goat (Santa Cruz Biotechnology) or goat anti-rabbit (GE Healthcare, Buckinghamshire, England) IgG for 2 hours at room temperature. Antibody-labeled proteins were detected using an enhanced chemiluminescence detection system (PIERCE, Rockford, IL). hJagged-1 or Dll-1 proteins was detected only in each transduced HESS-5 cell line, but not in the empty vector-transduced control (Fig 1B).

### Luciferase assays

Luciferase reporter assays were performed as described previously [20]. In brief, 2.5 × 10^5^ NIH/3T3 cells stably expressing Notch1 or 8 × 10^5^ CHO cells stably expressing Notch2 (provided by Dr. Hozumi K. Tokai University, Kanagawa, Japan) were transfected with RBP-Jk-luc (five RBP-J-binding sites) and pTK-Renilla plasmids (constitutive expression of Renilla luciferase for transfection efficiency control) by Transfast Transfection Reagent (Promega, Madison, WI). At 1 day after transfection, the cells were collected, and 5 × 10^4^ of cells were co-cultured with 5 × 10^4^ of each transduced HESS-5 stromal cell line for 48 hours. Then, luciferase activities were measured using a Dual Luciferase Kit (Promega) according to the manufacturer’s instructions. hJagged-1- and hDll-1-transduced HESS-5 cells were activated by RBP-Jk, a major Notch target. Control HESS-5 cells were not activated (n = 3) (Fig 1C).

### Co-culture assays

To evaluate the effects of Notch ligand-expressing stromal cells on the progeny of EPCs, the human EPC fraction of CD133^+^ CB cells, which mostly overlap with CD34^+^ cells, were plated onto the various stroma. Briefly, at 24 hours before co-culture experiments, hJagged-1, hDll-1, and control HESS-5 cells (1 × 10^4^ in 0.5 mL medium) were plated in flat-bottomed, collagen-I-coated 24-well plates. CD133^+^ CB cells (1 × 10^4^) were plated onto irradiated HESS-5 cell layers in 24-well plates containing Stem Span SFEM (Stem Cell Technologies, Vancouver, BC, Canada) supplemented with 5% fetal bovine serum (FBS) (JRH Bioscience, Lenexa, KA) and cytokines including human stem cell factor (SCF) (100 ng/mL), interleukin (IL)-6 (20 ng/mL), thrombopoietin (TPO) (20 ng/mL), Flt-3 ligand (100 ng/mL), and vascular endothelial growth factor (VEGF) (50 ng/mL). Human SCF, IL-6, and TPO were a generous gift from Kirin Brewery Co. Ltd. (Tokyo, Japan). Flt-3 ligand and VEGF were purchased from PeproTech (London, UK). All cytokines were pure human recombinant molecules. At 7 days after incubation at 37°C with 5% CO_2_, the cells were harvested, counted, and used for subsequent analyses and experimental assays. In some cultures, 10 μg/mL γ-secretase inhibitor IX (GSI IX: Calbiochem, Darmstadt, Germany) was added to the culture medium to inhibit Notch signal transduction.

### Reverse transcription-polymerase chain reaction (RT-PCR) analysis

Total RNA was obtained from cultured CD133^+^ CB cells using an RNeasy Micro Kit (QIAGEN GmbH, Hilden, Germany) according to the manufacturer’s instructions. First-strand DNA was synthesized from 100 ng RNA with random primers by a First Strand cDNA Synthesis Kit (Invitrogen) and amplified with specific primer pairs by Taq DNA polymerase (Takara, Otsu, Japan). The human specific primer pairs, PCR conditions, and products sizes are shown in S1 Table. Human umbilical cord vein endothelial cells were used as a positive control. PCR products were visualized in 2% ethidium bromide-containing agarose gels. To quantify vasculogenic gene expression in cultured CD133^+^ human CB cells, we measured the band intensities in gel images. After the images were recorded in a computer, the band intensities were processed with Image J software (http://imagej.en.softonic.com/). Specific mRNA expression levels were normalized to the intensities of human glyceraldehyde-3-phosphate dehydrogenase (hGAPDH).

### EPC colony-forming assay

Vasculogenic methylcellulose culture for the EPC colony-forming assay was performed in triplicate for each sample as described previously [21]. Briefly, 1 mL aliquots of a culture mixture containing 5 × 10^2^ cultured CD133^+^ CB cells, Iscove’s modified Dulbecco’s medium (IMDM) (Gibco), Methocult H4236 (Stem Cell Technologies), 30% FBS (JRH Bioscience), penicillin/streptomycin, and cytokines, including human SCF (100 ng/mL), VEGF (50 ng/mL), basic fibroblast growth factor (FGF) (50 ng/mL), epidermal growth factor (EGF) (100 ng/mL), insulin-like growth factor (IGF)-1 (100 ng/mL), IL-3 (20 ng/mL), and heparin (2 IU/mL), were plated in 35-mm Primaria culture dishes (BD Falcon, Franklin Lakes, NJ). All cytokines were pure human recombinant molecules. Human IL-3 was a generous gift from Kirin Brewery Co. Ltd. Basic FGF, EGF, IGF-1 (PeproTech), and heparin (LEO Pharma, Ballerup, Denmark) were purchased. Cultures were incubated at 37°C with 5% CO_2_ for 18 days, and then the number of cells was counted by microscopy. For flow cytometric analysis and the cell adhesion assay, EPC colonies were stained with EPC fluorescent chemical markers [22]. Briefly, EPC colonies were incubated with 2 μg/mL DiI-labeled acetylated low density lipoprotein (DiI-Ac-LDL) (Biomedical Technologies, Stoughton, MA) at 37°C for 1 hour. Methylcellulose was removed, and the cells were washed twice with PBS to remove non-adherent colonies. The remaining attached EPC colonies were harvested by pipetting 2 mM EDTA in PBS. For flow cytometric analysis, EPC colonies were fixed with 1% paraformaldehyde for 10 minutes and then reacted with 10 μg/mL FITC-labeled *Ulex europaeus* lectin type 1 (UEA-1) (Vector Laboratories Inc., Burlingame, CA) for 1 hour at 4°C, followed by flow cytometry. For the cell adhesion assay, stained and harvested cells from EPC colonies were counted, and then 2 × 10^4^ cells were incubated at 37°C in 0.1% BSA/IMDM with 100 ng stromal derived factor-1 (PeproTech) on 0.1% gelatin-coated (Sigma) 24-well plates. After 20 minutes, non-adherent cells were removed by washing with PBS, and adherent cells were counted by fluorescence microscopy.

### Transplantation of EPCs experienced with Notch ligands into ischemic hindlimb *in vivo*

All experimental procedures were conducted in accordance with the national and institutional guidelines. The protocols were approved by the Institutional Animal Care and Use Committee of the Isehara Campus, Tokai University School of Medicine, based on the Guide for the Care and Use of Laboratory Animals (National Research Council). Sixteen male athymic nude mice (CLEA Japan, Inc., Tokyo, Japan), 8 to 9 weeks old and 17 to 20 grams in weight, were separated into four groups (Control, hJagged-1, hDll-1 and IMDM) and used. Unilateral hindlimb ischemia was induced in the mice by ligating and excising the left femoral artery, as described previously [23, 24]. All animals were housed in a room under a 12-h light and 12-h dark cycle and controlled humidity and temperature, with free access to food and water. The experimental animal protocols for making ischemic models were performed under adequate anesthesia using 1.5% to 2.0% isoflurane (Dainippon Sumitomo Pharma Co., Ltd., Osaka, Japan) to minimize pain in the mice in line with the 3Rs (replacement, reduction and refinement). After surgery, the mice were monitored twice a day and subcutaneously injected with buprenorphine (Repetan, 0.1 mg/kg body weight; Otsuka Pharmaceutical Co., Ltd., Tokyo, Japan) once a day for 3 days to relieve pain or discomfort. At sacrifice, pentobarbital sodium (Somnopentyl, 100 mg/kg body weight; Kyoritsu Seiyaku Co., Ltd., Tokyo, Japan) was intraperitoneally injected.

Cultured CB CD133^+^ cells were harvested and washed three times with a sufficient amount of IMDM to remove the culture medium completely. Then, 1 × 10^5^ cells in 50 μL of fresh unused IMDM were injected into the ischemic limb muscle of the mice immediately after the ligation procedure. As additional control animals, mice with hindlimb ischemia were identically injected with only medium of IMDM.

### *In vivo* physiological and histological assessment

Regional blood flow in ischemic hind limbs was recorded and analyzed by laser Doppler perfusion imaging (LDPI) at 4, 7, 14 and 28 days after transplantation as described previously [15]. In the digital color-coded images, the red hue indicated regions of maximum perfusion, while medium perfusion levels were shown as yellow and low levels as blue. The resulting images also displayed absolute values in readable units. For quantification, the ratio of readable units was determined between ischemic and nonischemic hind limbs. All mice were euthanized at 28 days after transplantation by intraperitoneal administration. Our protocol included humane endpoints in cases in which food or water could not be consumed. However, these were not required in any cases and there were also no deaths prior to the experimental endpoint. Vascular density in sections from the ischemic hind limbs was evaluated at the microvascular level using a fluorescence microscope. Tissue sections from the lower calf muscles of ischemic limbs were obtained on day 28. Muscle samples were fixed with 4% paraformaldehyde at 4°C, embedded in OCT compound (Sakura Finetechnical, Tokyo, Japan), snap-frozen in liquid nitrogen, and cut into 5 μm-thick sections. Histological staining with isolectin B4 (Vector Laboratories) was performed, and capillary density was evaluated morphometrically by histological examination of 15 randomly selected fields. To detect transplanted human cells in mouse ischemic limb muscles, immunohistochemistry was performed with antibodies against human leukocyte antigen (HLA)-ABC (BD Biosciences) and human von Willebrand factor (vWF) (DAKO, Carpinteria, CA). First, HLA-ABC and vWF were labeled with a Zenon® Alexa Fluor® 594 Mouse IgG1 Labeling Kit and then an Alexa Fluor®488 Mouse IgG2a Labeling Kit (Molecular Probes, Karlsruhe, Germany), and then the labeled antibodies were applied for 2 hours. Nuclear counterstaining was performed with 4′-6-diamidino-2-phenylindole (DAPI; Vector Laboratories).

### Statistical analysis

Statistical analysis was performed using StatView v5.0 (Abacus Concepts Inc., Berkeley, CA). All values are expressed as the mean ± standard deviation (SD). Statistical significance was evaluated by one-way analysis of variance. Differences of P < 0.05 were considered statistically significant.

## Results

### Effect of Notch ligands on CD133^+^ cell numbers

**Fig 2:**
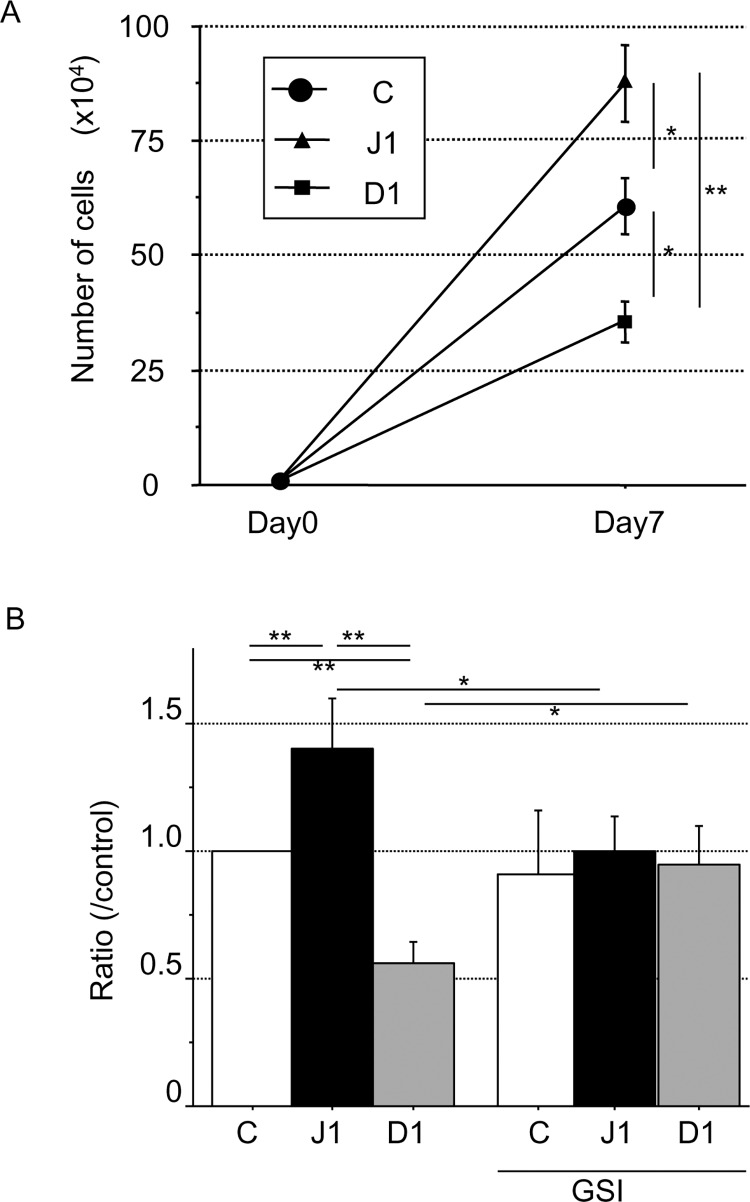
Effect of hJagged-1 and hDll-1 expression in HESS-5 stromal cells on nuclear cell proliferation. CD133^+^ CB cells (1 × 10^4^) were co-cultured with control (C), hJagged-1 (J1)- or hDll-1 (D1)-expressing HESS-5 stromal cells. (A) Expansion of nuclear cells at 7 days after co-culture. Data are expressed as means ± SD. **P < 0.01, *P < 0.05 between the indicated values. (B) Expanded cell numbers were normalized to cell numbers activated by control HESS-5 stromal cells at day 7. Data are expressed as means ± SD (n = 7 without GSI; n = 4 with GSI). **P < 0.01, *P < 0.05. GSI: γ-secretase inhibitor.

Numbers of hJagged-1-stimulated CD133^+^ cells significantly increased to 8.76 ± 2.07 × 10^5^ (P < 0.05) compared with control cells (6.09 ± 1.50 × 10^5^), whereas the number of hDll-1-stimulated CD133^+^ cells was reduced to 3.54 ± 1.07 × 10^5^ (P < 0.05) (Fig 2A). The expansion ratio to the control was 1.44-fold for hJagged-1-stimulated cells and 0.58-fold for hDll-1-stimulated cells. After blocking Notch signaling with GSI, the expansion ratios were similar to the control levels (n = 7 without GSI; n = 4 with GSI) (Fig 2B). No proliferation was observed in non-contact or contact co-culture with GSI (data not shown). Thus, hJagged-1 induces the expansion of CD133^+^ cells, whereas hDll-1 attenuates expansion.

### Effect of Notch ligands on EPC phenotypic differentiation

**Fig 3:**
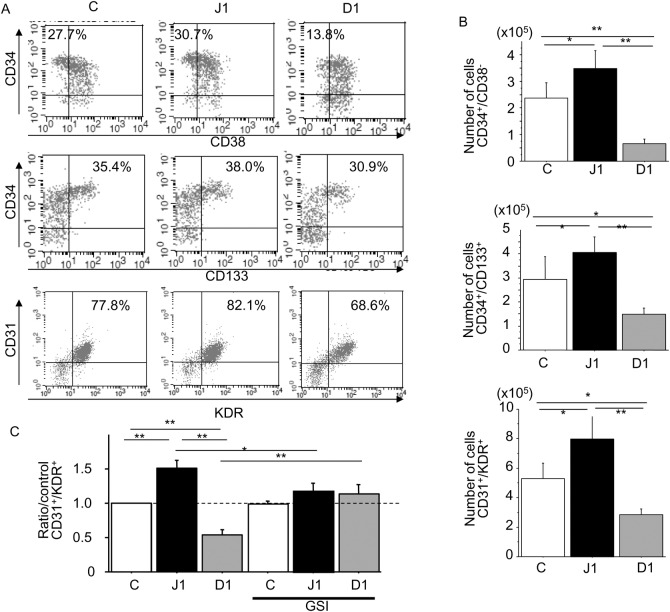
Effect of hJagged-1- or hDll-1-expressing HESS-5 stromal cells on the differentiation of CD133^+^ CB cells. (A) Flow cytometric analysis of CD31, CD34, CD38, CD133, and KDR expression on CD133+ cells co-cultured with control (C), hJagged-1 (J1)- or hDll-1 (D1)-expressing HESS-5 stromal cells at day 7. Numbers in the graph indicate the percentages of gate events within the quadrant. (B) CD34^+^/CD38^-^, CD34^+^/CD133^+^, and CD31^+^/KDR^+^ cell numbers in 1 × 10^4^ CD133+ cells at 7 days after co-culture with the three types of HESS-5 stromal cells. Data are expressed as means ± SD (n = 5). **P < 0.01, *P < 0.05 between the indicated values. (C) Expanded cell numbers were normalized to the cell numbers activated by control HESS-5 cells at day 7. Data are expressed as means ± SD (n = 5 without GSI; n = 3 with GSI). **P < 0.01, *P < 0.05 between the indicated values. GSI: γ-secretase inhibitor.

We next evaluated the effects of hJagged-1 and hDll-1 on the maintenance of EPCs and commitment to the endothelial lineage. Phenotypic analysis of collected cells co-cultured with stromal cells by flow cytometry revealed that the percentages of CD34^+^/CD38^-^ hematopoietic progenitor cells and CD31^+^/KDR^+^ endothelial lineage cells were decreased among hDll-1-stimulated CD133^+^ cells (Fig 3A). The percentages of CD34^+^/CD38^-^, CD34^+^/CD133^+^, and CD31^+^/KDR^+^ cells among hJagged-1-stimulated CD133^+^ cells were increased slightly, but not significantly compared with the control (Fig 3A).

The numbers of CD34^+^/CD38^−^ hematopoietic progenitor cells and CD34^+^/CD133^+^ immature EPCs were (P < 0.05) increased significantly among hJagged-1-stimulated CD133^+^ cells compared with the control (Fig 3B). Furthermore, the numbers of CD31^+^/KDR^+^ endothelial linage cells were increased significantly by 1.5-fold among hJagged-1-stimulated CD133^+^ cells (P < 0.01), but were decreased significantly by 0.5-fold among hDll-1-transfected CD133^+^ cells (P < 0.01). Suppression of Notch signaling by GSI reversed these cellular changes compared with the controls (n = 5 without GSI; n = 3 with GSI) (Fig 3C).

**Fig 4:**
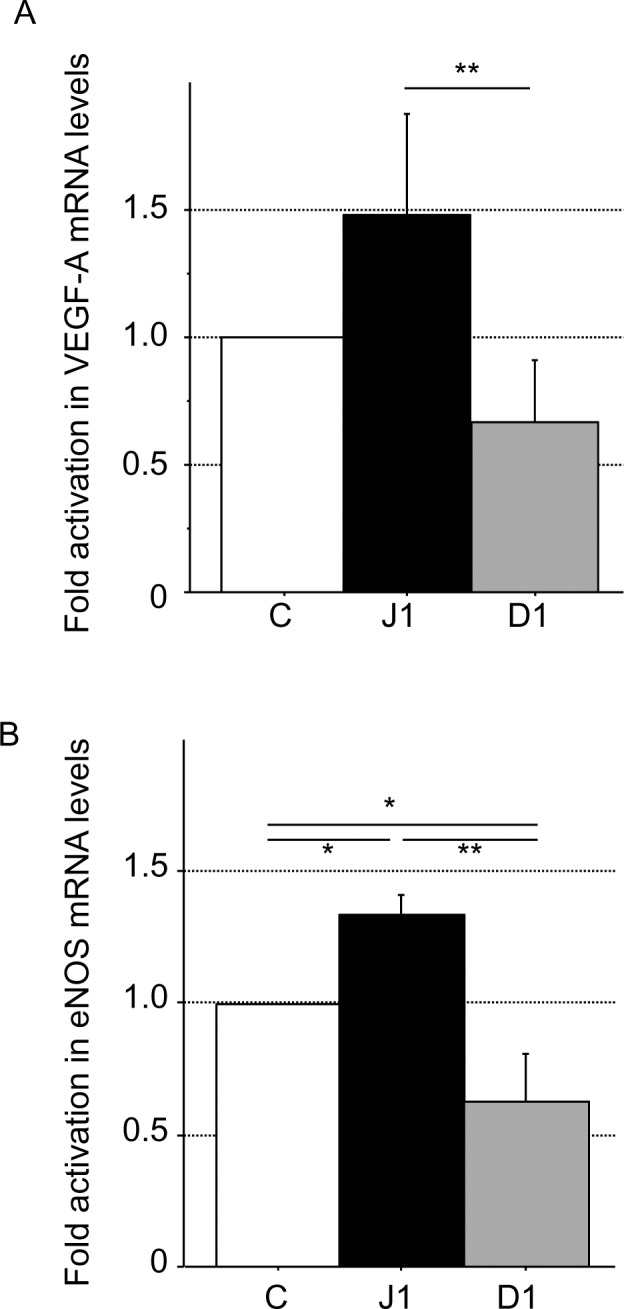
Expression analysis of a human endothelial marker and angiogenic factor by RT-PCR. Expression of human endothelial marker and angiogenic factor mRNAs by competitive RT-PCR analysis of CD133^+^ cells co-cultured with control (C), hJagged-1 (J1)- or hDll-1 (D1)- expressing HESS-5 stromal cells. Expression of VEGF-A (A) and eNOS (B) mRNAs were normalized to the mRNA expression level activated by control HESS-5 stromal cells at day 7. Data are expressed as means ± SD (n = 3). **P < 0.01, *P < 0.05 between the indicated values.

To confirm the EPC phenotype of Notch ligand-stimulated cells, we evaluated the expression of VEGF-A and endothelial nitric oxide synthase (eNOS) mRNAs by competitive RT-PCR analysis. All cell types examined expressed VEGF-A and eNOS mRNA. There was significantly higher VEGF-A and eNOS mRNA expression in hJagged-1-stimulated CD133^+^ cells compared with Dll-1-stimulated CD133^+^ cells (both P < 0.05) (Fig 4). HESS-5 stromal cells expressing hJagged-1 or hDll-1 differentially affected the proliferation and differentiation of CD133^+^ cells to EPC lineages. Thus, Jagged-1 and Dll-1 have opposite effects on the emergence of EPCs.

### Effect of Notch ligands on EPC colony formation

**Fig 5:**
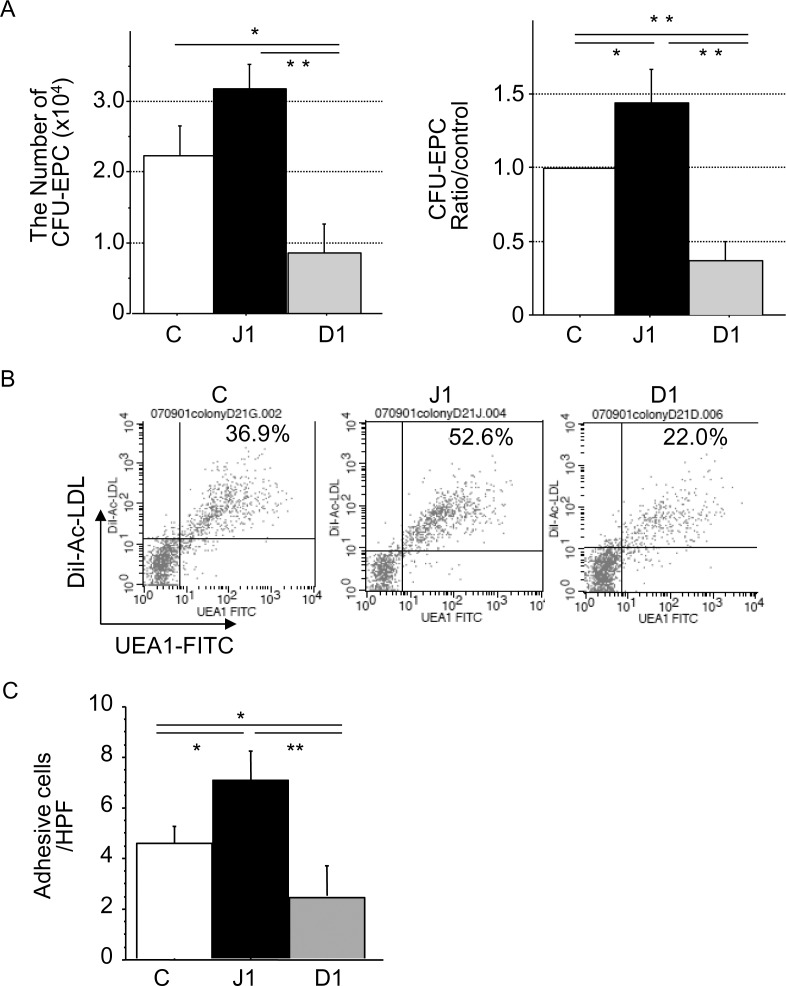
Formation and properties of EPC colonies. Formation and properties of EPC colonies derived from CB CD133^+^ cells co-cultured with control (C), hJagged-1 (J1)- or hDll-1 (D1)-expressing HESS-5 stromal cells. (A) Numbers of EPC colony forming units (EPC-CFU) were determined at 18 days after co-culture. Data are expressed as means ± SD (n = 3). **P < 0.01, *P < 0.05 between the indicated values. Flow cytometric analysis of DiI-Ac-LDL uptake and UEA-1-FITC expression as markers of EPCs (B) and the adhesive ability (C) of EPC colonies derived from 5 × 10^2^ CD133^+^ CB cells stimulated with each HESS-5 stromal cell type for 7 days. Numbers in the graph indicate the percentages of gate events within the quadrant of DiI-Ac-LDL+/UEA-1+. Data are expressed as means ± SD (n = 3). **P < 0.01, *P < 0.05 between the indicated values.

Isolated CD133^+^ cells were stimulated with each stromal cell type at day 7 for EPC colony forming assays. EPC colonies expressed endothelial differentiation markers KDR, eNOS, and VE cadherin (data not shown). Compared with controls, the number of EPC colonies were significantly higher in hJagged-1-stimulated CD133^+^ cell cultures (P < 0.05) and significantly lower in hDll-1-stimulated CD133^+^ cell cultures (P < 0.05) (Fig 5A). Compared with controls, EPC colonies derived from hJagged-1-stimulated CD133^+^ cells were enriched with Ac-LDL/UEA-1 double-positive cells and showed significantly increased adhesive activity (P < 0.05), whereas hDll-1-stimulated cells showed a lower adhesive activity (Fig 5B and 5C).

Thus, HESS-5 bone marrow stromal cells expressing hJagged-1 enhanced EPC colony formation of CD133^+^ cells.

### Transplantation of hJagged-1-stimulated CD133^+^ cells into mouse ischemic hindlimbs

**Fig 6:**
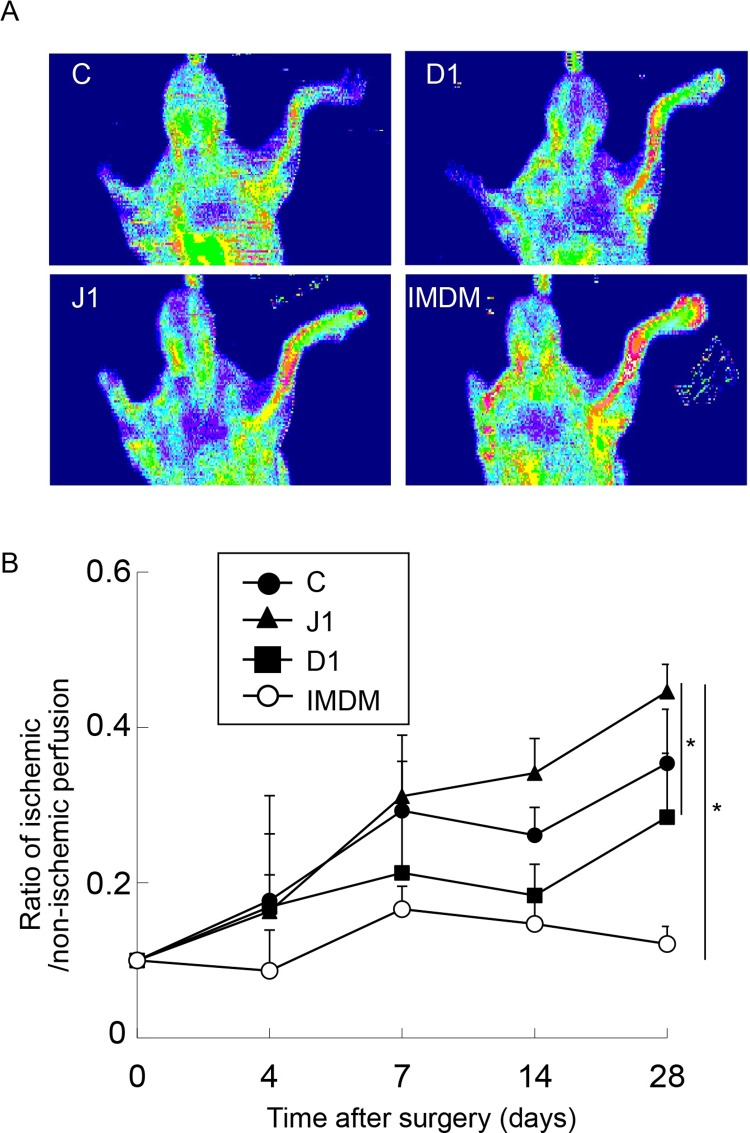
*In vivo* effects of transplanted EPCs stimulated by Notch ligands in mouse ischemic hindlimbs. CD133^+^ cells (1 × 10^5^) co-cultured with control (C), hJagged-1 (J1)- or hDll-1 (D1)-expressing HESS-5 stromal cells for 7 days or fresh unused medium only (IMDM) were injected into ischemic limb muscles immediately after femoral artery and vein ligation. LDPI was performed before and at days 4, 7, 14 and 28. (A) Representative LDPI images of each group at day 28. (B) Color-coded recordings were analyzed by calculating the mean perfusion for each foot (ischemic and non-ischemic) from day 0 to 28. Perfusion is expressed as the ratio of left (ischemic) and right (non-ischemic) hindlimbs. Data are expressed as means ± SD (n = 4 in each group). *P < 0.05 between the indicated groups.

**Fig 7:**
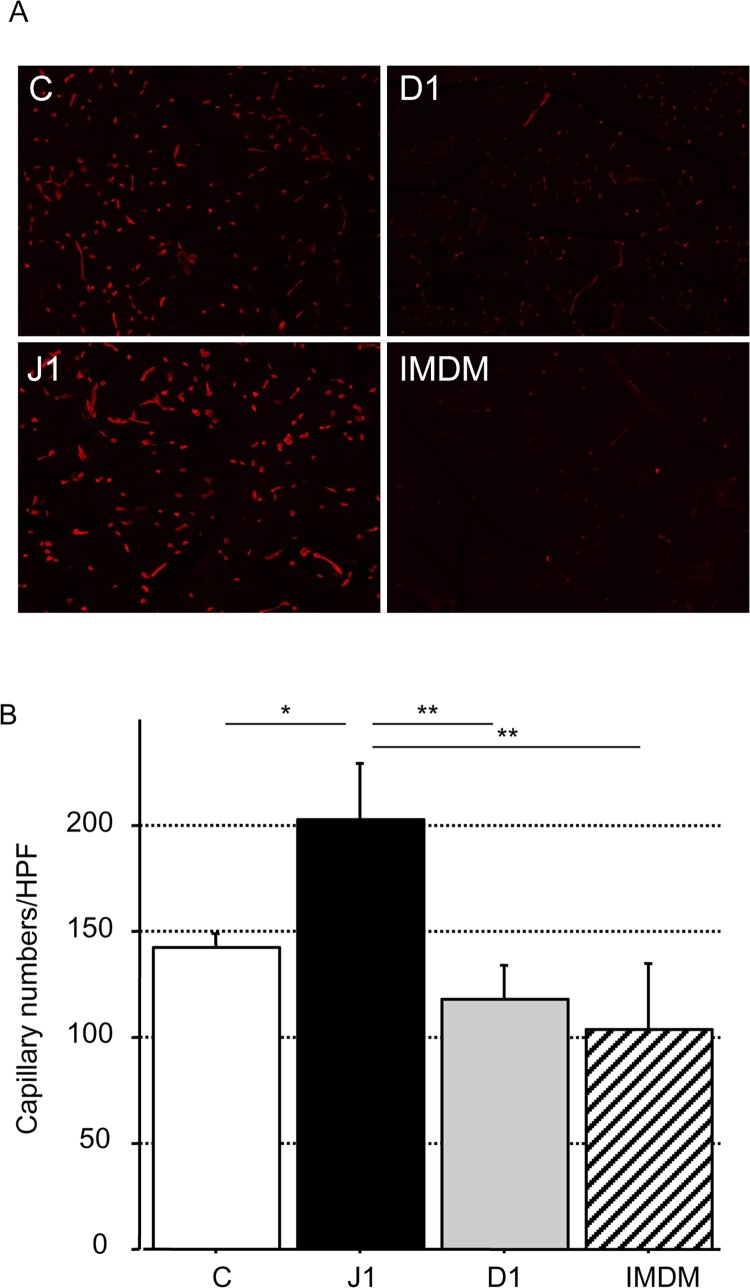
Histological evaluation of neovascularization at day 28 after EPC transplantation. (A) Representative isolectin B4 staining (red) in each group at day 28 after transplantation (×100 magnification). (B) Capillary density in hindlimbs injected with CD133^+^ CB cells (1 × 10^5^) co-cultured with control (C), hJagged-1 (J1)- or hDll-1 (D1)-expressing HESS-5 stromal cells or medium only (IMDM). Data are expressed as means ± SD (n = 4 in each group). **P < 0.01, *P < 0.05 between the indicated groups.

Serial examination of hindlimb perfusion by LDPI was performed at several time points from day 0 to 28. Representative images of each group at day 14 are shown in Fig 6A. Regional blood flow in ischemic hindlimbs of mice was increased after transplantation of the three types of stimulated CD133^+^ cells. Blood flow was significantly increased at 28 days after injection of hJagged-1-stimulated CD133^+^ cells compared with hDll-1-stimulated cells or control cells (P < 0.05) (Fig 6B). Capillary density in ischemic hindlimbs of mice was significantly higher after transplantation of hJagged-1-stimulated cells compared with hDll-1-stimulated cells or control cells (P < 0.05) (Fig 7A and 7B).

### Differentiation of transplanted cells stimulated by Notch ligands in ischemic muscles *in vivo*

Human endothelial cells derived from transplanted CD133^+^ cells co-cultured with Notch ligand-transduced stromal cells were identified in the vasculature by double staining of human HLA-ABC and human vWF. Human endothelial cells were identified in mice that received all three cell types, indicating that the transplanted human CD133^+^ cells had differentiated into endothelial cells in mouse ischemic muscles (S1 Fig).

## Discussion

This study demonstrated that bone marrow stromal cell environments expressing hJagged-1 or hDll-1 have different effects on CD133^+^ CB cell proliferation and differentiation into EPCs *in vitro* and *in vivo*.

Hemangioblasts in developing embryos and adults are common precursor cells for hematopoietic and endothelial cells, and express cell surface markers such as CD34, CD133, and Tie-2 [25, 26]. Nevertheless, although many investigators have expanded hematopoietic stem/progenitor cells *ex vivo* by bone marrow niche Notch signaling, the results are controversial. The apparent discordant biological activities of soluble Notch ligands in liquid and solid phase hematopoiesis have been assessed [27]. It was established that soluble Notch ligands exert greater biological activities under solid-phase conditions by immobilizing signaling antibodies. While the mechanisms of the different activities of Notch ligands in liquid and solid phases are unclear, soluble ligands are not physiological products and the immobilized form might function similarly to the membrane-bound forms. To reproduce the bone marrow microenvironment *in vitro* with cytokines, we used membrane-bound forms of Notch ligands on HESS-5 stromal cells that have the reconstituting ability of *ex vivo*-generated human hematopoietic stem cells. Our system was considered feasible as an *in vivo* bone marrow niche compared with previously reported systems [28].

To investigate whether these phenotypic and functional differences were associated with differences in Notch ligand signal transduction, Delaney et al. demonstrated dose-dependent effects of Dll-1 on the growth and differentiation of hematopoietic cells [29]. In this study, we demonstrated that, although human *Jagged-1*- and human *Dll-1-*transduced HESS-5 stromal cells activated RBP-Jk Notch transcription factors at similar levels, the proliferation and differentiation of CD133^+^ cells stimulated by each stroma were different. These results suggested that the different effects of Jagged-1 and Dll-1 on EPCs were not caused by the dose-dependency of Notch signaling, but rather the different roles of Jagged-1 and Dll-1.

Targeted mutagenesis and transgenic studies have reported the specific roles of Notch receptors as well as Jagged and Delta ligands [30–32]. Although Notch signaling has different roles in embryonic vascular development of mutant mice, most mutants exhibit a similar phenotype characterized by the absence of angiogenic vascular remodeling *in utero* and abnormalities of arterio-vein differentiation [30–32]. Notch signaling, such as Jagged-1/Notch4, Dll-1, and RBP-Jk, have important roles in embryogenesis and the post-natal vasculature [33, 34]. Sainson et al., showed that the Notch pathway regulates blood vessel sprouting and branching in adult endothelial tip cells, and high expression of Jagged-1 in these tip cells [35]. Buchler et al., suggested up-regulation of tumor angiogenesis by Jagged-1/Notch1 and Dll-4 [36]. Thus, Notch signaling plays multiple roles during vascular development and post-natal angiogenesis. However, some of these roles are controversial. It is thought that specific combinations of Notch ligands and receptors might be involved in different aspects of endothelial cell biology [37], and each Notch signal might influence other Notch signals as lateral inhibition [38]. We have demonstrated that downregulation of mouse Jagged-1 significantly reduces EPC commitment in bone marrow and impairs blood vessel regeneration by decreasing EPCs [15]. In this study, hJagged-1 expressed in bone marrow microenvironmental stromal cells induced the proliferation and differentiation of EPCs. These results suggest that the Jagged-1/Notch axis is critical for EPC development in the bone marrow niche.

Jagged-1 and Dll-1 pathways might be different in EPCs, although the underlying mechanisms remain to be elucidated. It is possible that different Notch receptors or different intracellular signal pathways are present in EPCs. Notch signaling is initiated by interactions between Notch receptors and their ligands on cells. Four Notch receptors (Notch 1–4) have been identified in mammalian systems. Previously, we found that CD133^+^ CB cells, which are similar to CD34^+^ cells, express all Notch receptors [6]. It has been reported that Notch1 signaling is involved in development and postnatal angiogenesis, although its role in EPCs is unclear [39]. Among the intracellular signaling pathways, mitogen-regulateactivated protein kinase and phosphatidylinositol 3-kinase/protein kinase B pathways are downstream of Notch signals for the proliferation of endothelial cells [40]. Choi et al., demonstrated that inhibition of glycogen synthase kinase-3β enhances EPC proliferation [41]. Not only ligand-specific signals but also expressional profiles of receptor/ligand on EPCs are additionally discussed. It is indicated that Dll-1 stimulation may down-regulate Jag-1 expression or signaling via a negative feedback effect on Notch receptors, possibly lateral inhibition [35], while Jagged-1/Notch3 and Dll-1/Notch1 positive feedback systems were reported [37, 42]. Thus, the identification and characterization of receptor/ligand and intracellular signaling pathways of Notch signaling that are crucial in EPC functions require further investigation.

VEGF is a potent promoter of angiogenesis and regulator of blood vessel homeostasis. Furthermore, VEGF contributes to postnatal neovascularization by mobilizing EPCs [12]. In previous studies of mammalian endothelial cells, Notch signals were induced by downstream signals of the VEGF receptor [43]. In contrast, it was recently reported that Jagged-1 and Jagged-2 increase VEGF secretion, and that Notch1 activates hypoxia-inducible factor-1α, the main regulator of VEGF in cancer angiogenesis [36]. In the present study, VEGF expression was increased in EPCs stimulated by hJagged-1-expressing HESS-5 cells. Similar mechanisms of cancer angiogenesis might occur in the bone marrow niche and EPCs. Further studies are needed to clarify the relationship between Notch signals and VEGF in the bone marrow niche.

In this study, the transplantation of hJagged-1-stimulated EPCs recovered impaired regional blood flow and capillary density *in vivo* in mouse ischemic hindlimbs. Immunohistochemical analysis demonstrated that transplanted CD133^+^ CB cell-derived EPCs differentiated into endothelial cells in ischemic limb muscles, but a significant difference in the numbers of human endothelial cells was not found among the groups. It has been reported that the angiogenic potential of transplanted EPCs in ischemia is due to endothelial differentiation and the local production of angiogenic cytokines[14]. Thus, enhanced neovascularization induced by injection of hJagged-1-stimulated CD133^+^ CB cells might be caused by an enhancement of EPC potential (colony formation and release of angiogenic factors including VEGF). Although the proliferation rate was different, we obtained the largest number of *ex vivo*-expanded EPCs from hJagged-1-stimulated CD133^+^ cells. Thus, hJagged-1 signaling in the bone marrow niche might be effective to enhance the therapeutic potential of CD133^+^ CB cell-derived EPCs.

In conclusion, this study is the first report regarding the effect of human Notch ligand signaling on EPC proliferation and differentiation. hJagged-1-expressing bone marrow-derived stromal cells induced the proliferation and differentiation of CD133^+^ CB progenitors compared with hDll-1-expressing cells. Thus, hJagged-1 signaling in the bone marrow niche might aid the expansion of EPCs for therapeutic angiogenesis.

## Supporting Information

S1 Fig Histological evaluation of human endothelial cell development *in vivo*. CD133^+^ CB cells (1 × 10^5^) co-cultured with control (C), hJagged-1 (J1)- or hDll-1 (D1)-expressing HESS-5 stromal cells for 7 days or fresh unused medium only (IMDM) were injected into mouse ischemic limbs. Representative double immunofluorescence staining of human vWF (red), HLA-ABC (green), and nuclear counterstaining with DAPI (blue) are shown for each group at 28 days after transplantation. All images show merged staining at ×400 magnification. Human endothelial cells were identified as vWF and HLA-ABC double-positive yellow cells (arrow). Scale bar represents 20 μm.
(TIF)

S1 Table Sequences of PCR primers for hVEGF-1, heNOS and hGAPDH, product sizes, and PCR conditions. (DOCX)
