# Supplementary figures and images for: Jagged-1 Signaling in the Bone Marrow Microenvironment Promotes Endothelial Progenitor Cell Expansion and Commitment of CD133+ Human Cord Blood Cells for Postnatal Vasculogenesis
Source: PLoS One. 2016 Nov 15;11(11):e0166660. doi: 10.1371/journal.pone.0166660 (PMC5112804; doi:10.1371/journal.pone.0166660)

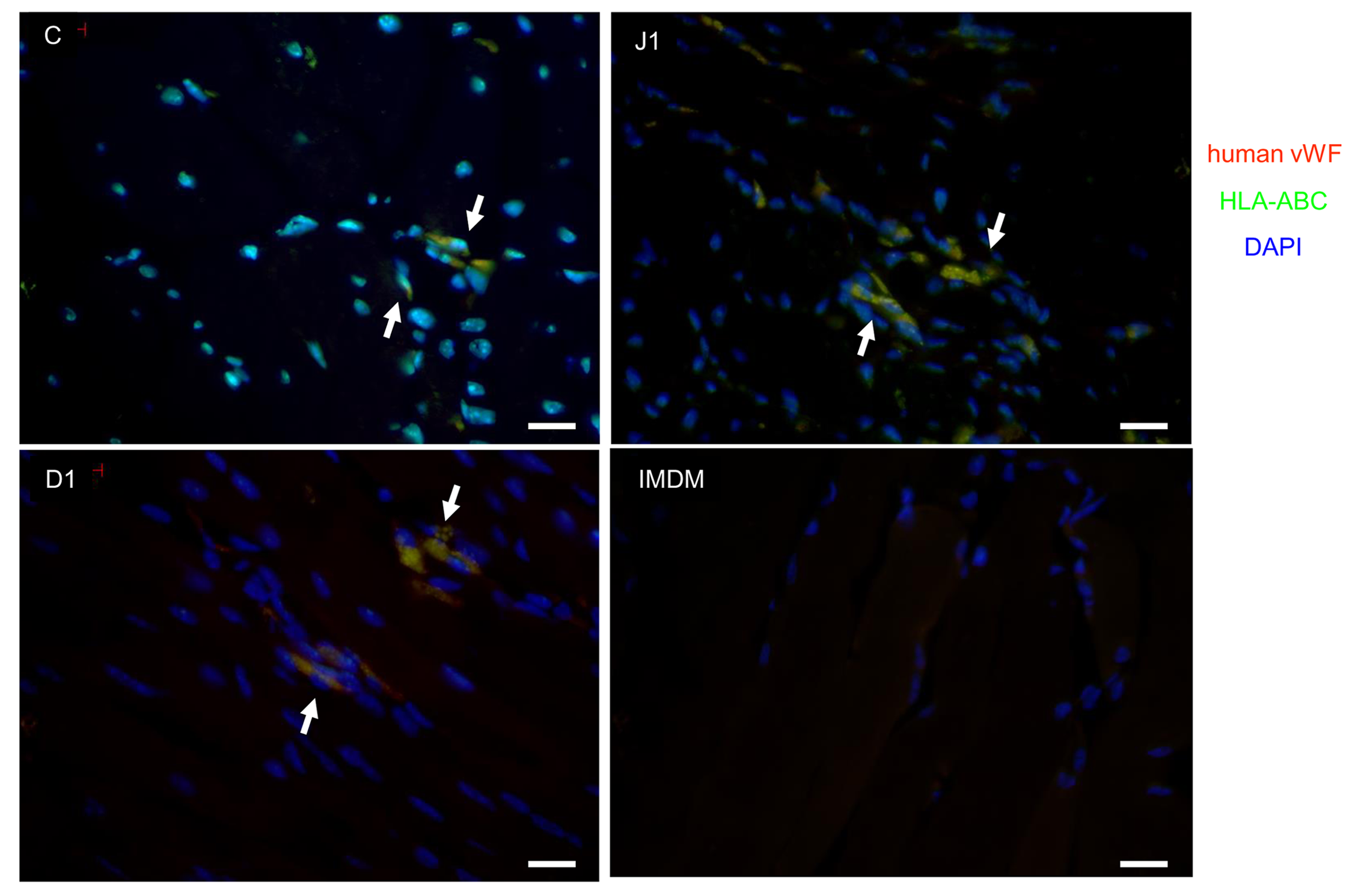

Supplement: S1 Fig — CD133+ CB cells (1 × 10^5^) co-cultured with control (C), hJagged-1 (J1)- or hDll-1 (D1)-expressing HESS-5 stromal cells for 7 days or fresh unused medium only (IMDM) were injected into mouse ischemic limbs. Representative double immunofluorescence staining of human vWF (red), HLA-ABC (green), and nuclear counterstaining with DAPI (blue) are shown for each group at 28 days after transplantation. All images show merged staining at ×400 magnification. Human endothelial cells were identified as vWF and HLA-ABC double-positive yellow cells (arrow). Scale bar represents 20 μm. (TIF) [file pone.0166660.s001.tif]
