# Supplementary material for: Jagged-1 Signaling in the Bone Marrow Microenvironment Promotes Endothelial Progenitor Cell Expansion and Commitment of CD133+ Human Cord Blood Cells for Postnatal Vasculogenesis
Source: PLoS One. 2016 Nov 15;11(11):e0166660. doi: 10.1371/journal.pone.0166660 (PMC5112804; doi:10.1371/journal.pone.0166660)
Supplement: S1 Table — (DOCX) [file pone.0166660.s002.docx]

**Table S1.** Sequence of PCR primers for hVEGF-1, heNOS and hGAPDH, product size, and PCR conditions.

| Target mRNA | Forward Primer  Reverse Primer | PCR product  Annealing/Cycle |
| --- | --- | --- |
| hVEGF-A | 5′-GTCGCACTGAAACTTTTCGTCCA-3′ | 541 bp  56°C/30 cycle |
|  | 5′-CAGTAGCTGCGCTGATAGACATCCA-3′ |  |
| heNOS | 5′-AACCACATCAAGTATGCCACCAACC-3′ | 455 bp  64°C/40 cycle |
|  | 5′-CGTGCCGATCTCAGTGCTCA-3′ |  |
| hGAPDH | 5′-CTGATGCCCCCATGTTCGTC-3′ | 596 bp  64°C/20 cycle |
|  | 5′-CACCCTGTTGCTGTAGCCAAATTCG-3′ |  |

PCR, polymerase chain reaction; hVEGF-A, human vasculogenic endothelial growth factor A; heNOS, human endothelial nitro oxidase synthetase.
